# 12 weeks high intensity interval training versus moderate intensity continuous training in chronic low back pain subjects: a randomised single-blinded feasibility study

**DOI:** 10.1186/s40945-022-00136-3

**Published:** 2022-05-02

**Authors:** Tamara Cerini, Roger Hilfiker, Thomas F. Riegler, Quinten T. M. Felsch

**Affiliations:** 1 Department of Physiotherapy, Schulthess Klinik, Zurich, Switzerland; 2 Department of Physiotherapy, University of Applied Sciences and Arts Western Switzerland Valais (HES-SO Valais-Wallis), Leukerbad, Switzerland; 3 Department of Physiotherapy, University of Applied Sciences of Zurich (ZHAW), Winterthur, Switzerland; 4 Department of Sports Medicine, Schulthess Klinik, Zurich, Switzerland

**Keywords:** Chronic low back pain, Non-specific low back pain, Endurance, Training, High intensity interval training, Moderate intensity continuous training

## Abstract

**Background:**

Currently, very little is known about the effects of an endurance high intensity interval training (HIIT) in chronic low back pain patients. Therefore, the feasibility and safety of the HIIT must be assessed first before Currently, very little is known about the effects of an endurance high intensity interval training in chronic low back pain patients. Therefore, the feasibility and safety of the HIIT has to be assessed first before it can be integrated safely into research and daily practice it can be integrated safely into research and daily practice. This study aims to answers the question if high intensity interval training and moderate intensity continuous training (MICT) have comparable adherence and feasibility.

**Methods:**

Participants (age from 29 to 69 years) with non-specific chronic low back pain were recruited in this randomised, single-blinded, allocation concealed, feasibility study. The participants trained 30 min on a cycle ergometer for 12 weeks. One group had HIIT and the other MICT.

**Results:**

Of 45 screened subjects 30 participated. The adherence rate was 94% in the HIIT group (median 0.94, IQR 0.23) versus 96% in the MICT group (median 0.96, IQR 0.08), without between-group differences: estimated median of the difference of − 0,01 [95% CI, − 0.11 to 0.06; *p* = 0.76]. Similar results in enjoyability (median 3, IQR 1 vs median 2, IQR 1.8) and willingness to continue the training (median 3, IQR 1 vs median 3, IQR 0.4).

Both groups improved in pain and disability, without between-group differences in pain [median of the difference, 0.5; 95% CI, − 1 to 2; *p* = 0.95] nor in disability [median of the difference, 1.78; 95% CI, − 6.44 to 9.56; *p* = 0.64].

**Conclusion:**

There were no differences in adherence rates. HIIT is as feasible as MICT in non-specific chronic low back pain and can be used in future larger trials to deepen the knowledge about HIIT in this specific population.

**Trial registration:**

ClinicalTrials.gov, NCT04055545. Registered 13 August 2019.


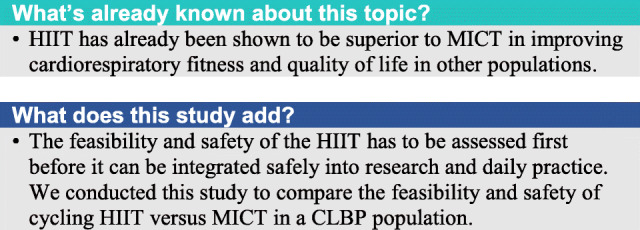


## Key message

Our data suggest that high-intensity interval training (HIIT) is as feasible and safe as moderate continuous training (MICT) for persons with chronic low back pain.

## Introduction

Chronic low back pain (CLBP) is one of the most common causes of musculoskeletal pain in the adult population. It is among the main reasons for disability worldwide [1]. Current guidelines for the treatment of non-specific low back pain recommend a conservative approach with exercise therapy as first line treatment [2, 3]. Endurance and resistance training are two examples of possible exercise therapies. Similar therapeutic effects have been reported between the different types of training therapies and adherence seems to be an important outcome factor [4]. Although no exercise therapy has shown to be superior to the other, it has been suggested that moderate to higher training intensities could be more effective in improving pain and disability in the chronic low back pain population [5].

Moderate intensity continuous training (MICT) is an established and recommended exercise therapy, used to increase cardiovascular function and reduce pain in CLBP [3, 4, 6]. So far it has been shown that high intensity interval training (HIIT) is effective in improving pain and disability in other health problems [7, 8]. HIIT has already been shown to be superior to MICT in improving cardiorespiratory fitness and quality of life and similar in enhancing vascular function [9, 10]. Currently, very little is known about the effects of an endurance high intensity interval training in chronic low back pain patients. Therefore, the feasibility and safety of the HIIT has to be assessed first before it can be integrated safely into research and daily practice. Future studies are necessary to evaluate efficacy and effectiveness.

We aimed to answer the question, if HIIT has a comparable adherence and feasibility to MICT?

## Method

### Design

We conducted a monocentric, randomized, allocation concealed, age-stratified, parallel-group, participant-blinded, feasibility study.

The recruitment was performed consecutively and the randomisation was stratified. The age stratification sequence was created by an external researcher not involved in the study. The cut-off was set at 49 years, to ensure a fair age distribution. To guarantee allocation concealment the research team did not have access to the randomisation list. An allocation ratio of 1:1.1 resulted from the age-stratified randomisation. The randomisation and data collection were managed using the web-based Research Electronic Data Capture System (REDCap) [11].

We conducted this study to compare the feasibility and safety of 12 weeks cycling HIIT versus 12 weeks cycling MICT in a CLBP population. High intensity interval training and moderate intensity continuous training on a cycle ergometer were chosen as the endurance interventions to compare. Each group trained for the same amount of time thirty minutes, three times a week for three months.

The participants were blinded and not aware about the group allocation. In addition, they were not informed about the differences of the exercise modalities.

### Participants, therapists, centres

All participants were screened and recruited in the Schulthess Clinic in Zurich by rheumatologists, orthopaedic surgeons, sport physicians and physiotherapists. The subjects gave written informed consent. Participants had 7 days to decide about their participation after receiving the study information sheet.

Eligibility criteria were checked a second time by the principal investigator for safety purposes. Participants were eligible if between 29 and 69 years of age, had low back pain for at least 3 months and had an Oswestry disability index (ODI) score of at least 14% [12]. We chose 14% to be comparable with other low back pain (LBP) studies as for recruitment purposes [13–15]. A good understanding of German or English (written and spoken) was mandatory. Before entering the study, the referring physician was consulted for clearance to perform a steep ramp test to determine peak heart rate, to start above mentioned exercise regimes and if at least one answer of the Physical Activity Readiness Questionnaire (PAR-Q) was checked as yes. Inclusion was only allowed if there was no or stopped physical therapy at the start of the study.

Exclusion criteria were pre-existing unstable heart disease or suspected angina pectoris, cardiac dysrhythmias, heart failure, aneurysm or aortic stenosis; previous low back spinal surgery in the last 2 years; spinal stenosis, spinal fractures, tumour or radiculopathy; diabetes mellitus, rheumatoid arthritis or other systemic inflammatory diseases or known pregnancy.

### Intervention

Training protocols and fitness test methods were selected according to the American College of Sport Medicine’s (ACSM) guidelines [16].

The intervention consisted of two different supervised training protocols (HIIT vs MICT) on a cycle ergometer (Proxomed®, Kardiomed® 530, Steckborn, Switzerland). Every participant had to maintain 70 rpm (RPM) for the entire duration of the cycle program. Training sessions were separated by at least 24 h of rest. The training supervisor was the same for all participants for the entire duration of 12 weeks for each participant.

#### Steep ramp test

All the participants performed a steep ramp test on a cycle ergometer to assess their heart rate peak and watt peak. This fitness test was necessary to set the initial training watt and the target heart rate (THR) to reach the defined intensities for the HIIT or MICT protocol.

The heart rate (HR) and the rating of perceived exertion (RPE) were monitored during the steep ramp test. Each participant started with 3 min warm up at 40 watts. The workload was increased every 3 s for 1 watt till exhaustion or if the RPM fell under 50. The result of the test was used to adapt the HIIT and the MICT to the subject’s current performance state using the heart rate reserve (HRR) method to find the THR.

#### HIIT protocol

The participants allocated to the HIIT group started with a five-minute warm up, then performed 10 × 60 s burst followed by 60 seconds recovery after each burst. The training was concluded with five minutes of cool down. The burst phases had an intensity of > 90% of the HRR and the recovery phases between 30 and 39% [16].

#### MICT protocol

The MICT program also started with five minutes of warm up, followed by 20 min of moderate training with an intensity between 40 to 59% of the HRR. Five minutes of cool down concluded the training [16].

### Outcome measures

At baseline sociodemographic and health data, like age, work status, practiced sport, duration of the low back pain and secondary diagnoses, disability index, average pain in the last 3 weeks and number of other areas of pain were collected.

#### Primary outcome

The primary outcome was the difference in training adherence rate (after 12 weeks training program) between HIIT and MICT. The adherence rate was defined as the number of days a patient trained versus the total days, they were scheduled to train but were not present. We wanted to evaluate whether HIIT would have the same or a greater adherence rate than MICT.

#### Secondary outcome

Secondary outcomes were divided in feasibility and effectiveness outcomes. For the feasibility outcomes, enjoyability (measured on a Likert scale from − 3 to + 3) and willingness to continue the training (Likert scale from − 3 to + 3) were collected after each single training. Other feasibility outcomes were dropout rate, adverse events connected with the training and screened subjects in relation with the number of recruited patients. Safety was interpreted as no difference in number and severity of adverse events (AE) and severe adverse events (SAE) in comparison to MICT. For the effectiveness outcomes, the Oswestry disability index and the average low back pain in the last 3 weeks on the numeric rating scale (NRS) were collected at baseline and after 12 week [12].

### Data analysis

Descriptive statistics was used to describe the characteristics of the participants.

For the primary outcome, the Shapiro-Wilk-test was performed and resulted in a non-normal distribution. Therefore, the primary analysis was made with non-parametric statistic. The difference in adherence rate between groups was performed with the Mann-Whitney test and a non-parametric confidence interval with the estimated difference in location were calculated [17]. The difference between groups was described with median and interquartile range (HIIT vs. MICT). Intention to treat (ITT) and per protocol analysis were performed (PP) for the primary outcome.

The secondary outcomes were also analysed with non-parametric statistic. The Mann-Whitney test was performed to compare group (HIIT vs MICT) outcomes of pain, disability enjoyability and willingness to continue the training from baseline to the end of study. Intention to treat analyses was performed for the secondary outcomes.

Statistical analyses were performed using IBM Corp. (2019) IBM SPSS Statistics, Version 26.0. and R version 3.6.3 (2020-02-29) [18, 19].

No sample size was calculated. A sample size between 24 and 50 has been suggested for feasibility and pilot studies [20–22].

## Results

### Flow of participants through the study

**Fig. 1:**
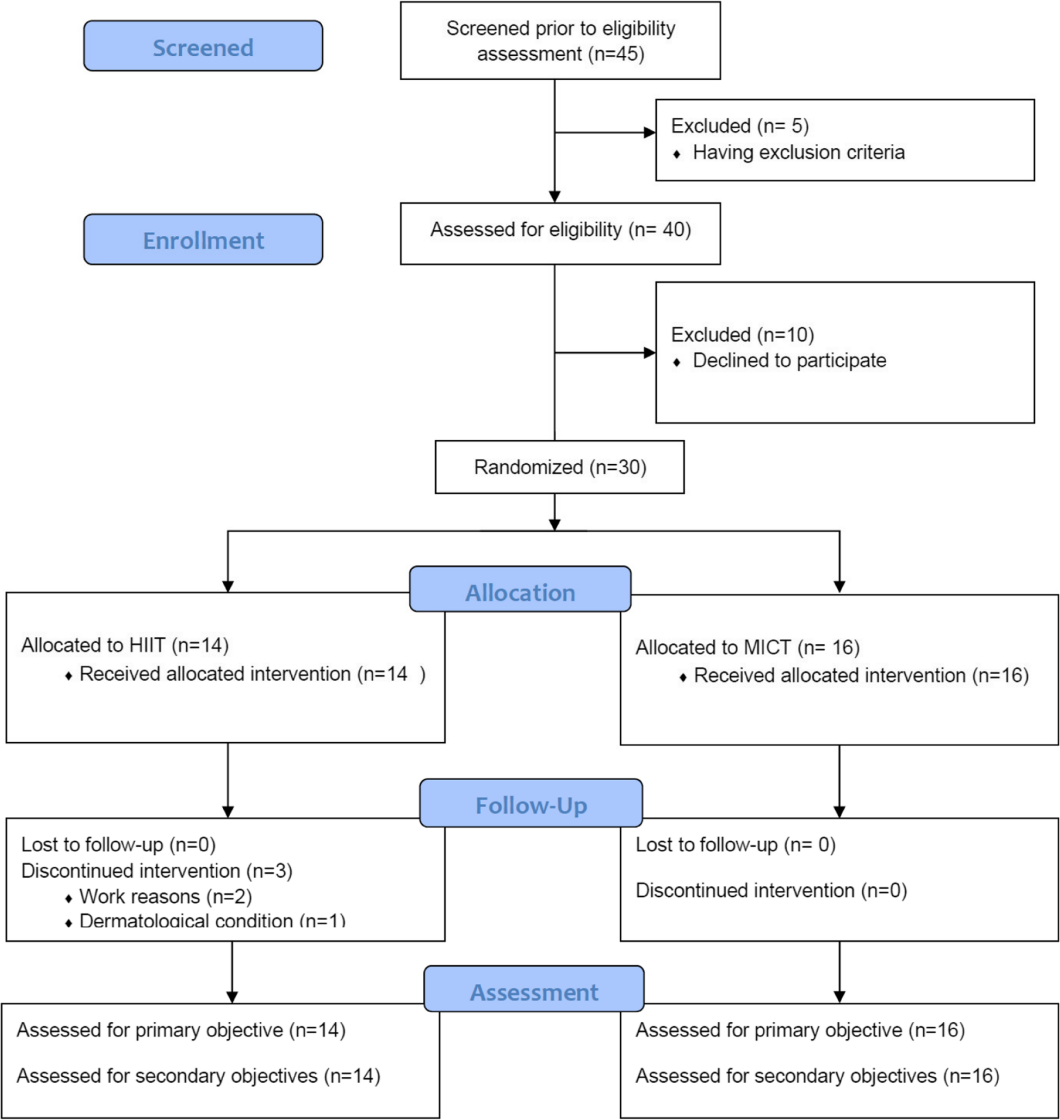
Flow of participants. According to the CONSORT statement for randomized pilot and feasibility trials [23]

45 individuals were screened between September and December 2019. 5 were not assessed for eligibility because having exclusion criteria. 2 of them were diagnosed with spinal stenosis and 3 had spinal surgery in the last 2 years. After being assessed for eligibility another 10 refused to participate. As shown in Fig. 1, 30 participants were eligible and included in the study. The baseline characteristics are described in Table 1. The groups were similar despite the small number of participants per group. The study ended in March with the last training of the last participant.

**Table 1:** Baseline characteristics

Baseline characteristics		
	HIIT (*n* = 14)	MICT (*n* = 16)
Age in years, Mean (SD)	50.29 (10.07)	50.44 (13.05)
Female sex, n (%)	8 (57.1)	8 (50)
Pain duration in months, Mean (SD)	122.71 (139.54)	89.31 (99.59)
Other pain sites, n (%)	11 (78.6)	10 (62.5)
Secondary diagnoses, n (%)	10 (71.4)	10 (62.5)
Smoking	1 (7.1)	4 (25)
Receiving pain medication, n (%)	7 (50)	8 (50)
Practicing sport, n (%)	10 (71.4)	12 (75.0)
Heart rate peak Mean (SD)	158.21 (22.306)	166.56 (13.276)
Watt peak Mean (SD)	195.57 (66.891)	218.56 (58.821)
Pain intensity last 3 weeks Mean (SD) ^a^	5.36 (2.061)	4.88 (1.628)
Oswestry disability index Mean (SD) ^b^	28.286 (10.8941)	25.438 (8.3175)

^a^ Numeric Rating Scale, from 0 (no pain) to 10 (worst pain possible). ^b^ Oswestry Low Back Disability Questionnaire, from 0% (minimal disability) to 100% (bed-bound)

### Primary outcome: adherence

The median (mdn) of the adherence rates of both groups were similar.

The MICT group had no dropout. The sixteen participants in this group performed 532 trainings out of 576. Fourteen participants were allocated in the HIIT group. Three discontinued the training, none of the reasons were related with the training. In the HIIT group the total of training sessions performed from the fourteen participants, were 422 out of 504. That results, in the ITT analysis, in an adherence rate of 94% (mdn 0.94, IQR 0.23) versus the MICT 96% (mdn 0.96, IQR 0.08) with an estimated median of the difference of − 0.01 [95% CI, − 0.11 to 0.06; *p* = 0.76]. The results showed that the HIIT group had a slightly smaller adherence than the MICT group, although not statistically significant.

Four participants in the HIIT group and 2 in MICT group were 100% adherent.

The PP analysis showed that the HIIT had a median of 0.97 (IQR 0.11) versus the MICT median of 0.96 (0.08) with an estimated median of the difference of 0.028 [95% CI, − 0.06 to 0.83; *p* = 0.45].

**Fig. 2:**
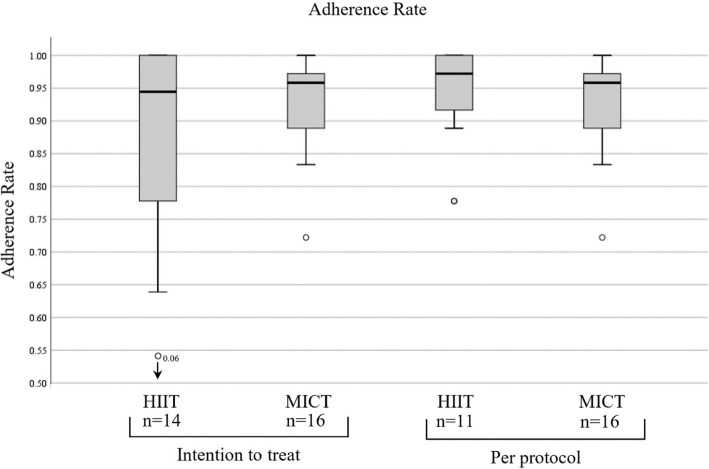
Adherence Rate

Figure 2 show the distribution of both groups in the “intention to treat” and “per protocol” analysis.

### Secondary outcomes: feasibility

**Fig. 3:**
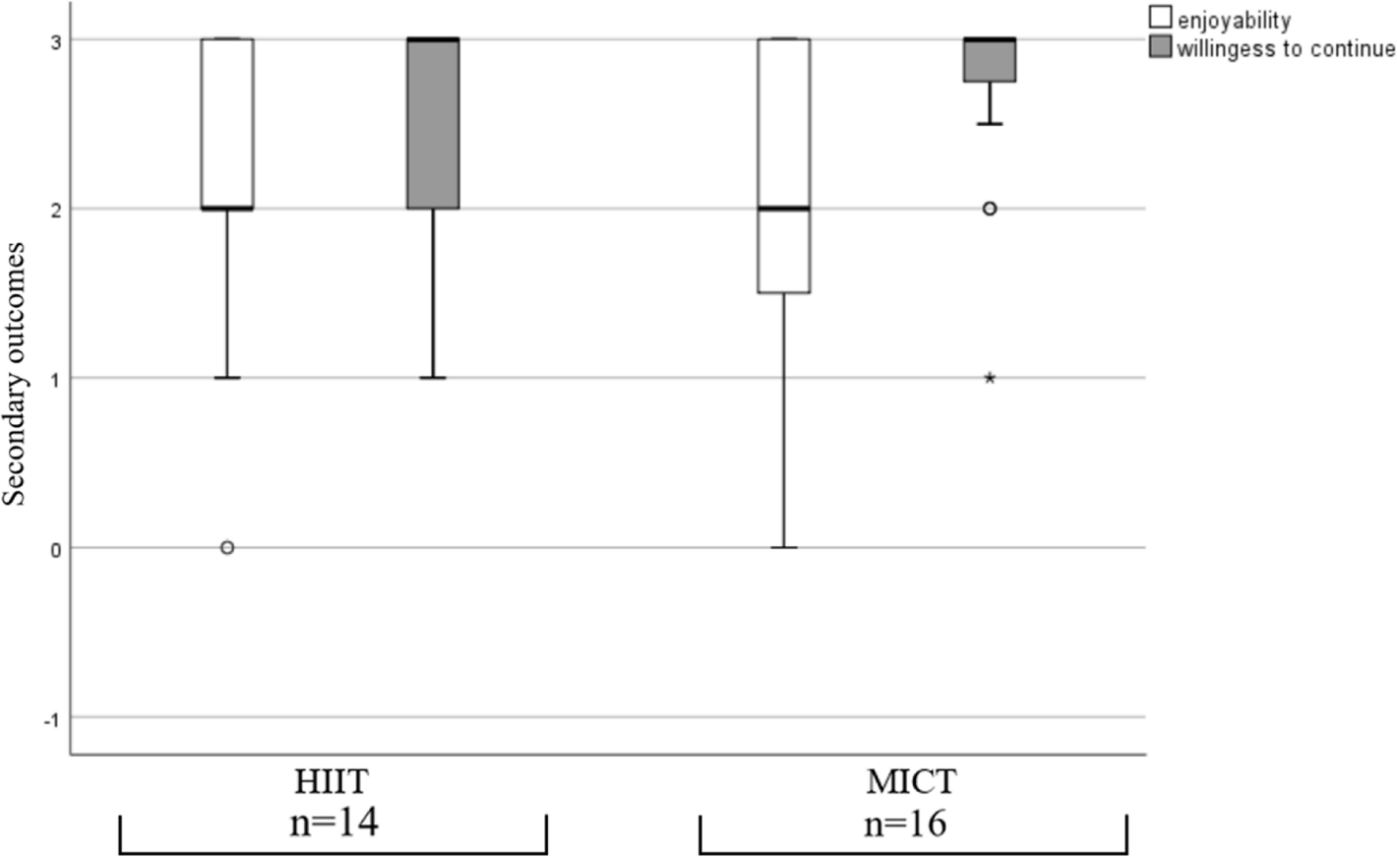
Secondary endpoints enjoyability and willingness to continue the training

There were no significant differences between the groups in the feasibility endpoints enjoyability and willingness to continue the training (Table 2). In Fig. 3 the distribution of both groups is shown for enjoyability and willingness to continue the training.

**Table 2:** Feasibility endpoints

Feasibility endpoints			
	HIIT (*n* = 14)	MICT (*n* = 16)	*p*-value
Enjoyability, median (IQR)	2 (1)	2 (1.8)	0.67
Willingness to continue the training, median (IQR)	3 (1)	3 (0.4)	0.42
Adverse events, n	8	9	
Persons reporting AE, n	4	4	

*p*-value from Mann-Whitney test comparing between-group differences

The screened subjects in relation with the number of recruited persons was 30/45. Meaning that 67% of the screened individuals could participate and were included in the trial.

The dropout rate was 0% in the MICT group and 21% in the HIIT group. Meaning that 3 participants of the HIIT group decided to discontinue the training. Two of the participants reported an increase of workload in their jobs leading to the discontinuation and the third had to stop due to a dermatological operation.

No serious adverse events occurred during the trial.

Other adverse events were reported in both groups. Four participants from the HIIT and four from the MICT reported the exact same kind of discomforts. These were muscle soreness and cramps in the legs, cervical or thoracic pain and knee pain. They reported the AE for a total of 8 times in the HIIT group and 9 times in the MICT group.

None of the participants discontinued the trial due to the reported AE. There was no necessity to stop or modify any of the training sessions of the eight participants due to the reported adverse events.

### Secondary outcomes: effectiveness

Both training groups had a significant decrease in pain and disability at the end of study after 12 weeks of training (Table 3).

**Table 3:** Effectiveness endpoints

Effectiveness endpoints			
	HIIT (*n* = 14)	MICT (*n* = 16)	*p*-value (between groups)
Oswestry disability index, baseline, median (IQR)	26 (18)	26 (13.25)	0.67
Oswestry disability index, 12 wk., median (IQR)	18 (16)	21(14.61)	0.64
*p*-value (within subjects)	0.009	0.003	
Pain intensity last 3 weeks, Baseline, median (IQR)	6 (3)	5 (2)	0.45
Pain intensity last 3 weeks, 12 wk., median (IQR)	3 (2)	3 (2)	0.95
*p*-value (within subjects)	0.003	0.001	

*p* value from Mann-Whitney test

The HIIT group reported a pain intensity median of 6 (IQR 3) at baseline and decreased to a median of 3 (IQR 3) at 12 weeks. The median of the the pain intensity in the MICT group was 5 (IQR 2) and decreased to a median of 3 (IQR 2). There were no between-group differences in pain [median of the difference, 0.05; 95% CI, − 1 to 2; *p* = 0.94].

A reduction in disability was also observed. The HIIT group started with a median of 26 (IQR 18) at baseline and improved to 18 (IQR 16) at the end of the study. The MICT group had a median of 26 (IQR 13.25) at baseline and improved to 21 (IQR 14.61) at the end of the study. There were no between group differences in disability [median of the difference, 1.78; 95% CI, − 6.44 to 9.56; *p* = 0.64].

## Discussion

The primary objective of this study was to compare the adherence rates between two endurance protocols (HIIT and MICT) in non-specific chronic low back pain. Our study indicates that HIIT and MICT have similar adherence rates. The HIIT group had four participants with an adherence rate of 100% versus two in the MICT group. Figure 2 shows there are more participants distributed closer to an adherence rate of 100% as in the MICT group, where most participants over the median are nearer to the median than the 100%. Under the median there is a wider distribution in the HIIT group, where the MICT group is nearer to the median. These results include the three participants who dropped out. None of these three participants discontinued the trial due to reasons related to the training. Moreover, one of the participants discontinued the training after only two sessions of 36, having a strong impact on the distribution of the HIIT adherence, given the already small sample. These are the reasons why a “per protocol” analysis was appropriate in this trial. Figure 2 shows the HIIT programme without the dropouts had a higher adherence than the MICT. It also shows a smaller distribution where all the subject’s adherence rates are nearer to a higher median as the MICT group. However, the difference between groups was not significant even in the per protocol analysis. The results suggest that both HIIT and MICT have good adherence. Our high adherence results are similar to studies using comparable training protocols [24, 25]. Good adherence to protocol is important because it enables a precise collection and interpretation of results of interventional trials [26].

There were no serious adverse events. Minor adverse events were reported in both groups from the same number of participants in each group. Interestingly, the reported events were the same in both groups, such as muscle soreness. Muscle soreness can be interpreted either as an adverse event or an expected effect of a moderate to high training protocol. During this trial eight participants reported muscle soreness. Therefore, assessing it as adverse is important, because it is not less expected than other adverse events. These discomforts had no negative interaction with the adherence or the training. It appears that the training protocols were equally safe for our sample.

Enjoyability and willingness to continue the training were also similar. Our data suggests that there is no difference between the two groups, neither in enjoyability nor in willingness to continue the training. The results about enjoyability, showed differences to other studies with similar training protocols. In other trials HIIT resulted in a better enjoyability than the moderate continuous trainings [27]. The HIIT protocol used in this trial is suggested to be one of the more feasible and enjoyable protocols (10 × 60 sec.) [28]. Despite this we found no differences between the HIIT and MICT. This could mean that for our sample, the training protocol was not relevant to achieve a high enjoyability. Therefore, as already suggested in the chronic low back pain guidelines, the choice of the exercise training modality should be chosen in accordance to the patient’s preferences [3]. In this case, it could be either HIIT or MICT.

After 12 weeks of training both groups had a significant decrease in pain and disability without differences between the groups. The minimal clinical important differences (MCID) recommended to use in CLBP is 2.4 points for the NRS and 17 points for the Oswestry disability index [29]. As described in Table 3, both our groups had a similar improvement in pain and disability. However, the only outcome showing a clinical important difference is the pain in the HIIT group, with a difference of 3 points from baseline (mdn 6) after 12 weeks (mdn 3). The HIIT group had a higher pain score at baseline. Therefore, the HIIT group had greater potential to show improvement compared to the lower pain in the MICT group. Literature suggests that higher training intensities could initiate physiological changes which could impact on inflammation markers and possibly on pain intensity [24, 30]. However, our results are a good representation of the complexity of chronic low back pain and the already well-established knowledge that this condition is not only biophysical. Therefore, an isolated physical exercise treatment is not the single answer to chronic low back pain, instead a bio-psycho-social approach is needed [31]. Future research investigating the efficacy and effectiveness of HIIT should add more patient specific and quality of life measures to ensure more significant outcomes for the chronic low back pain population. In the design of future trials, we also suggest carefully assessing the cut-off value of the ODI. In our study we chose 14% for recruitment purposes and to be comparable with other LBP studies [13–15], but it could be too low to measure clinical important differences.

This study has several strengths. To our knowledge it was the first trial assessing 12 weeks endurance HIIT program in CLBP and comparing it with MICT. The training supervisor was the same for every participant, ensuring that trainings were conducted the same way during the entire trial and minimizing possible differences of outcomes due to personal character preferences. Both training protocols used in this study were described by the ACSM’s guidelines [16]. This ensures a full replication of the protocols for future trials.

The study has also limitations. It was not possible to blind the care provider and the outcome assessor, because they were the same person. Because of the feasibility design and the absence of a sample size calculation, caution is needed in interpreting the results of the trial because it is not designed to evaluate treatment effects. Finally, the management of the THR in the HIIT group as described in the ACSM’s guidelines was very difficult. Due to slow adaption of the participants heart rates it was not possible to stay within the lower targeted range during the recovery phases [16]. However, THR during burst phases was achieved.

## Conclusion

In conclusion, our data suggest that HIIT is as feasible as MICT and can be used in future trials to investigate the effects of high intensity interval training in non-specific chronic low back pain.

## Data Availability

The datasets used and/or analysed during the current study are available from the corresponding author on reasonable request.

## Abbreviations

ACSM: American college of sport medicine
AE: Adverse event
CLBP: Chronic low back pain
HIIT: High intensity interval training
HR: Heart rate
HRR: Heart rate reserve
ITT: Intention to treat
LBP: Low back pain
MCID: Minimal clinical important difference
MICT: Moderate intensity continuous training
NRS: Numeric rating scale
ODI: Oswestry disability index
PAR-Q: Physical activity readiness questionnaire
PP: Per protocol
RPE: Rating of perceived exertion
RPM: Revolutions per minute
SAE: Severe adverse event
THR: Target heart rate
